# Supplementary material for: Introgression of the Powdery Mildew Resistance Genes Pm60 and Pm60b from Triticum urartu to Common Wheat Using Durum as a ‘Bridge’
Source: Pathogens. 2021 Dec 26;11(1):25. doi: 10.3390/pathogens11010025 (PMC8778237; doi:10.3390/pathogens11010025)
Supplement: Supplementary file 1 [file pathogens-11-00025-s001.zip › Figure S1-25-12-2021.pdf]

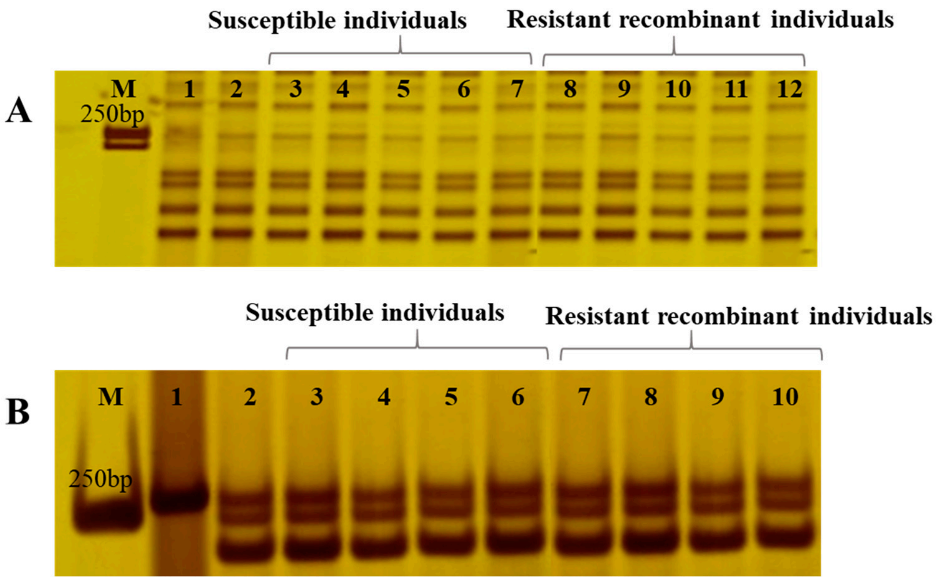

Figure S1. The genotypes by the markers which were linked to *Pm60* in the introgression lines. (A) Recombinant types screened for marker *Scaf32-5*.24: 1-2 are Mo75 and Xuezao, 3-7 susceptible individuals (Z2-1, Z2-2, Z2-5, Z2-12 and Z2-13), 8-12 resistant individuals (Z2-3, Z2-8, Z2-11, Z2-16 and Z2-19). (B) Recombinant type screened for *Scaf15-5.24*: 1-2 are Mo75 and Xuezao, 3-6 susceptible individuals (Z14-1, Z14-2, Z14-13 and Z14-14), 7-10 resistant individuals (Z14-3, Z14-7, Z14-8 and Z14-10).
